# Macrophage: Key player in the pathogenesis of autoimmune diseases

**DOI:** 10.3389/fimmu.2023.1080310

**Published:** 2023-02-14

**Authors:** Shuang Yang, Ming Zhao, Sujie Jia

**Affiliations:** ^1^ Dapartment of Dermatology, Hunan Key Laboratory of Medical Epigenomics, The Second Xiangya Hospital, Central South University, Changsha, Hunan, China; ^2^ Institute of Dermatology, Chinese Academy of Medical Sciences and Peking Union Medical College, Nanjing, China; ^3^ Key Laboratory of Basic and Translational Research on Immune-Mediated Skin Diseases, Chinese Academy of Medical Sciences, Nanjing, China; ^4^ Department of Pharmacy, Chinese Academy of Medical Sciences and Peking Union Medical College, Nanjing, China

**Keywords:** macrophage, systemic lupus erythematosus, rheumatic arthritis, systemic sclerosis, type 1 diabetes

## Abstract

The macrophage is an essential part of the innate immune system and also serves as the bridge between innate immunity and adaptive immune response. As the initiator and executor of the adaptive immune response, macrophage plays an important role in various physiological processes such as immune tolerance, fibrosis, inflammatory response, angiogenesis and phagocytosis of apoptotic cells. Consequently, macrophage dysfunction is a vital cause of the occurrence and development of autoimmune diseases. In this review, we mainly discuss the functions of macrophages in autoimmune diseases, especially in systemic lupus erythematosus (SLE), rheumatic arthritis (RA), systemic sclerosis (SSc) and type 1 diabetes (T1D), providing references for the treatment and prevention of autoimmune diseases.

## 1 Introduction

According to whether tissues and organs are targeted by the damaging immune response, autoimmune diseases classified into systemic autoimmune disease, such as systemic lupus erythematosus (SLE) and systemic sclerosis (SSc) and rheumatoid arthritis (RA), or organ-specific autoimmune diseases, such as thyroid disease, type 1 diabetes (T1D), myasthenia gravis and multiple sclerosis (1, 2). The autoimmune diseases are clinically diverse but share a fundamental etiology: the form of self-reactive antibodies, presence of self-reactive T cells, and activation of the innate immune system (3). Although the exact pathogenesis remains unclear, it is interesting to note that genetic, immunological, hormonal and environmental factors are important triggers for autoimmune diseases (4).

However, it is difficult to precisely inhibit the abnormal immunity activation triggered by pathogenic factors. The current treatment of autoimmune diseases is limited and relatively conservative, which mainly depends on the overall inhibition of the immune response. However, blindly suppressing the immune response can cause inevitable side effects such as infection. Therefore, there is an urgent need to understand the pathological mechanism that causes the initiation and development of autoimmune diseases so as to provide new ideas for the prevention and treatment of autoimmune diseases.

**Figure 1:**
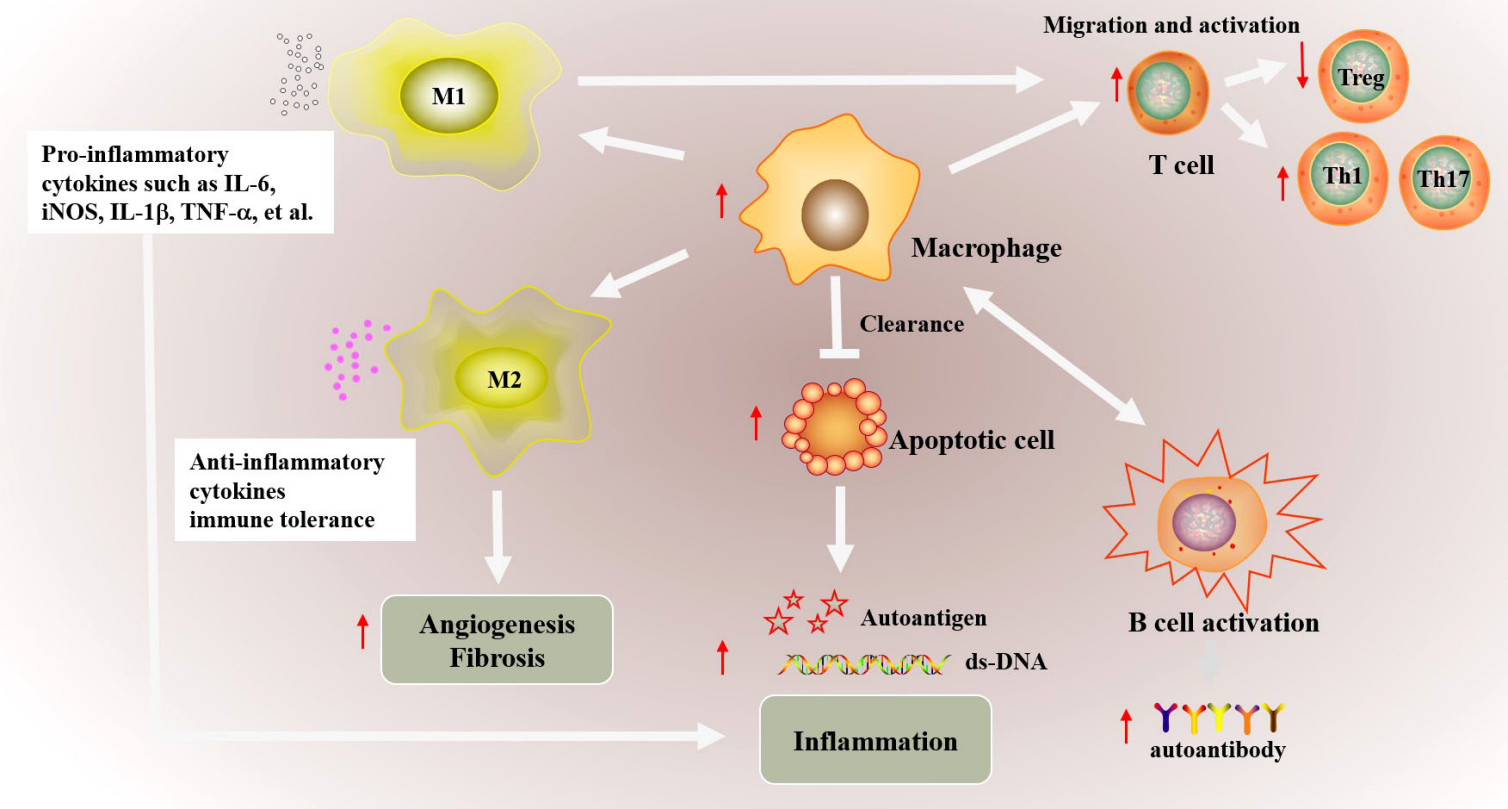
The possible abnormal macrophage activation in autoimmune diseases. The phagocytic function of macrophages is weakened in autoimmune diseases, which inhibits the clearance of apoptotic cells. Increased apoptotic cells promotes the production of autoimmune antigens and antibody, and further exacerbates inflammatory inflammation. In addition, macrophages promote the migration and abnormal activation of T cells including increased Th1/Th17 differentiation and downregulated Treg differentiation, and ultimately cause abnormal activation of B cells. Besides, the imbalance of M1/M2 macrophages also involved in autoimmune. Abnormal M1 macrophage activation promotes the production of proinflammatory cytokines such as IL-6, iNOS, TNF-α and IL-1β, which promote inflammation in targeted organs. Decreased M2 polarization inhibited the production of anti-inflammatory cytokines and the immune tolerance. Besides, abnormal M2 macrophage polarization also affects vascular proliferation, fibrosis in autoimmune disease such as SSc.

The innate immune system exerts immune function independently of antigens, which form the body’s immune defense system interacting with the adaptive immune system. Abnormal innate immune response is a significant reason for the breakdown of autoimmune tolerance, which is closely related to the occurrence and development of autoimmune diseases (5). Macrophage is a crucial part of the innate immune system and participates in almost every biological process such as tissue homeostasis, resisting infection, repairing after infection, metabolism and inflammation, affecting the body’s development and immune response (6, 7). This review summarizes the impaired functions and abnormal macrophage activation and their roles in the pathogenesis of autoimmune diseases showed in **Figure 1**, especially in SLE, RA, SSc and T1D. In addition, the potential value of macrophages in the treatment and prevention of autoimmune diseases is also summarized.

## 2 Macrophage

### 2.1 The origin of macrophages

It has been universally accepted that macrophages in tissues are differentiated from monocytes that originate in bone marrow (8). However, studies in recent years have found that monocytes are not the only source of macrophages. Tissue macrophages are also derived from the yolk sac and fetal liver, which have self-renewal properties independent of monocyte recruitment (9, 10). According to the tissue distribution, macrophages can be divided into alveolar macrophages, intestinal macrophages, osteoclasts in bone, microglia in the brain, Kupffer cells in the liver, Langerhans cells in the epidermis (10). Secondary lymphoid organs also have distinct macrophages, including marginal zone macrophages (MZMs) and metallophilic macrophages in the spleen, which involved in clearance of apoptotic cell and tolerance to auto-antigens (11). It is worth noting that microglia and partial Langerhans cells are derived from yolk sac progenitor cells as shown by pedigree tracing experiments. In contrast, macrophages in other tissues, such as intestinal lamina propria and dermis, are mainly derived from hematopoietic stem cells (12–16). Macrophages are, therefore, key tissue sentinel cells that react to tissue-specific signals, while retaining the ability to execute physiological functions such as phagocytes. During chronic inflammation such as autoimmune diseases, tissue-resident macrophages fail to solve aggravated inflammation that leads to immune system abnormal activation and damage. And peripheral monocytes are recruited and differentiated into macrophages non-homeostatically in combination with injury-associated signals including pro-inflammatory cytokines, which are further activated and participated in the body’s immune responses (17, 18). The tissue-resident macrophages participate jointly in protecting tissue homeostasis, and form the first line of defense against invading pathogens. Miriam. et al. considered that embryonically derived and monocyte-derived tissue-resident macrophages are likely to promote the development of the disease through the maintenance of tissue homeostasis through phagocytosis of cell fragments, resistance to pathogen invasion, while recruited monocyte-derived macrophages by disease-associated signals drives disease progression (19). Similarly, recruited monocyte-derived macrophage also plays an important role in autoimmune related diseases. For example, infiltrated macrophages, especially proliferating macrophages was seen in glomerulonephritis from patients with lupus, which may be a potential diagnostic and prognostic indicator for renal injury (20). Ly6C is a marker for circulating monocytes in mice. Different monocyte subpopulations Ly6C^hi^ and Ly6C^lo^ exist in mice, which express different adhesion molecule and chemokine receptor and gene expression profile. Response to inflammatory signals Ly6C^hi^ monocytes could rapidly infiltrate in inflamed tissues mostly dependent on chemokine receptors C-C motif chemokine receptor 2 (CCR2), CCR6 and CCR8 and results in enhanced liver fibrosis (21). Inhibiting migration of blood monocyte into liver alleviated macrophage infiltration in liver, and decreased pro-inflammatory cytokines such as interferon gamma (IFNγ), IL-6 expression in chronic hepatic injury (22). Besides, inhibiting monocyte recruitment by blocking C-C motif chemokine ligand 24 (CCL24) or CCL2 may be an appealing novel therapy to limit fibrotic manifestations of SSc (23).

The complex origin of macrophages has caused great difficulties in the study of macrophage functions in autoimmune diseases. Although the construction of mice with myeloid knockout has brought a lot of convenience for the study of macrophages *in vivo*, it also has certain limitations. On one hand, there are many kinds of myeloid cells, and it is difficult to accurately study the function of a single macrophage. On the other hand, macrophages in different tissue are heterogeneous and plastic, showing different morphologies and surface molecules. With the development of scientific research, especially in flow cytometry and single cell sequencing technology, the macrophage markers in different tissues are gradually discovered, and the research of macrophages ushers in new opportunities and challenges. At present, the studies about macrophage from different sources is limited, and mostly current studies focus on the abnormal function and mechanism of macrophages infiltrated in targeted organs and tissues. How to specifically distinguish unbalanced macrophages, specially manufacture macrophages that promote disease, and supplement and maintain tissue stable macrophages are the key and difficult points in autoimmune disease research.

### 2.2 Regulation in innate and adaptive immunity

Macrophages are vital participant of innate immunity, which recognize and effectively respond to invading pathogens, thus providing an early defense against external attack. Pattern recognition receptors (PRRs) on the surfaces of macrophage including toll-like receptors (TLRs) and the NOD-like receptors (NLRs) recognize pathogen-associated molecular patterns (PAMPs) and endogenous danger-associated molecular patterns (DAMPs) presented in the invaders and promote macrophage activation. Macrophages further release antimicrobial mediators to target the invading pathogen, chemokines to recruit immune cells to the inflammatory site, and pro-inflammatory cytokines to aggravate further inflammation, and even induce the adaptive immune response for the particular invading pathogen. Besides, macrophage forms a bridge connecting innate and adaptive immunity by presenting endogenous or exogenous antigen. It has been well known that antigen cross-presentation is crucial for initiating of adaptive immune responses against cancer, infection and immune tolerance. During this process, antigen-presenting cells (APCs) present intracellular and extracellular peptides derived from ingested antigens on primary histocompatibility complex class I (MHCI) protein complex to T lymphocytes (24). Although the cross-presentation of antigens by macrophages is not understood as well as that by dendritic cells (DCs), it is becoming clear that the cross-presentation by macrophages especially in spleen, liver and lymph nodes may help activate CD8^+^ T lymphocytes (25).

Macrophage can participate in antigen presentation to Th1 cells and proliferation of T cells by surface co-expression molecules CD86 and MHCII, which indicate the significant role of the macrophage in the development of cancer, autoimmunity and viral infections (26–29). The CD8^+^ T cells in mice with spontaneous autoimmune peripheral neuropathy (APN) exhibit an effector/memory phenotype required for the disease initiation. However, only effector/memory CD8^+^ T (CD8^+^ TEM) cells are not sufficient to induce autoimmune-mediated peripheral neuropathy and macrophages are additionally required (30). The early depletion of regulatory T cells (Tregs) in mice with acute cardiac injury enhances the inflammatory activation of macrophages by increasing the production of IFN-γ, which restrains muscle regeneration (31). Human macrophages activated by C1q can inhibit the T helper (Th)17 and Th1 but promote Treg proliferation, orchestrating the adaptive immune system to avoid autoimmunity (32). Hence, the role of macrophage in connecting innate/adaptive immunity provides opportunities to prevent disease onset, reduce relapses and develop new therapeutic strategies. Intervening macrophage-T cell communication signals to prevent excessive activation of T cells may be an important research direction in the treatment of autoimmune diseases

### 2.3 Phagocytic, efferocytosis and secretory functions

Phagocytosis is an essential process for the uptake of particulate matter, including microbes and dying cells. Dying cells can expose and secrete signals that attract phagocytes and promote their phagocytosis. Several studies have shown that macrophage phagocytosis is affected by a variety of signaling pathways including TLRs (26). Reactive oxygen species (ROS) generated by the nicotinamide adenine dinucleotide phosphate oxidases (NADPH oxidase-2, also known as NOX2) in macrophages is dispensable for phagocytosis (33). The liver X receptors (LXRs) and the peroxisome proliferator-activated receptors (PPARs), nuclear receptor families that regulate genes involved in lipid metabolism and transport are important components of macrophage phagocytosis (34). The phagocytosis of dead and dying cells is a process known as efferocytosis, which is performed by macrophages, other immune phagocytes such as monocyte and DCs and non-phagocytes including epithelial cells. Efficient efferocytosis limits the release of intracellular PAMPs that drive inflammation and disrupting homeostatic efferocytosis can also lead to accumulation of uncleared apoptotic cells in autoimmune diseases. Efferocytosis mechanisms depends on the signaling programs depicted: chemoattractant-mediated recruitment of phagocytes, receptor-mediated recognition such as PtdSer receptor cell immunoglobulin mucin receptor 4 (TIM4), TAM family receptor tyrosine kinase receptor, engulfment of apoptotic cells, and the processing of engulfed cellular material (35). Disrupted efferocytosis of macrophage promoted the accumulation of uncleared apoptotic or necroptosis cells in autoimmune, which is a universal feature of damaged tissues (36).

In response to exogenous danger signals or exogenous signals recognized by pattern-recognition receptors (PRRs), macrophages undergo physiological changes to initiate signal transduction cascades and result in abnormal production of chemokines, cytokines and toxic mediators, which can further enhance inflammation and contribute to autoimmune pathologies (37). The anti-inflammatory mediators by macrophages contribute to the dissolution of the inflammatory response. Cytokines such as tumor necrosis factor (TNF)-α, Interleukin (IL)-6, IL-1β, IL-12, IL-18, IL-23 and chemokines such as CXC chemokine ligands (CXCL)1, CXCL3 are secreted by macrophages, which are essential mediators and drivers of chronic inflammation and autoimmune diseases (38–40). Besides, macrophages contribute to angiogenesis by secreting proangiogenic proteases such as matrix metalloproteinases (MMP)-9 and MMP-12 (41–43). TNF-α occupies a pivotal position in RA pathogenesis. The TNF blockade reduced stromal cell activation, angiogenesis, and sustain regulatory pathways by mediating cytokine and chemokine and MMPs expression. And IL-6 signaling pathway promotes T cell activation and migration by regulating chemokine expression (44). In addition to clearing dead cells, macrophages significantly mediate wound healing and tissue homeostasis by producing anti-inflammatory molecules and tissue remodeling growth factors like IL-10 and transforming growth factor beta(TGF-β) (45). Cytokines including IL-6, IL-23, IL-10 and TGF-β all shaped Th17 cell differentiation placed at the center of autoimmune inflammation (46). IL-18 contributes to Th1/2 differentiation, participate in cytotoxic T cells (CTLs) and natural killer (NK) cells activation, and ultimately IgE production from B cells (47). Besides, tissue macrophages synthesize chemokines CXCL1/CXCL2 to increase neutrophil recruitment, which is an important early step in controlling tissue infections or injury (48). Islet-resident and islet-infiltrating macrophages can exacerbate β-cell destruction by synthesizing TNF-α, IL-12, IL-1β, and NOX2-derived ROS, which mature autoreactive CD4 and CD8 T cell effector responses (49).

### 2.4 Regulation in metabolic processes

**Figure 2:**
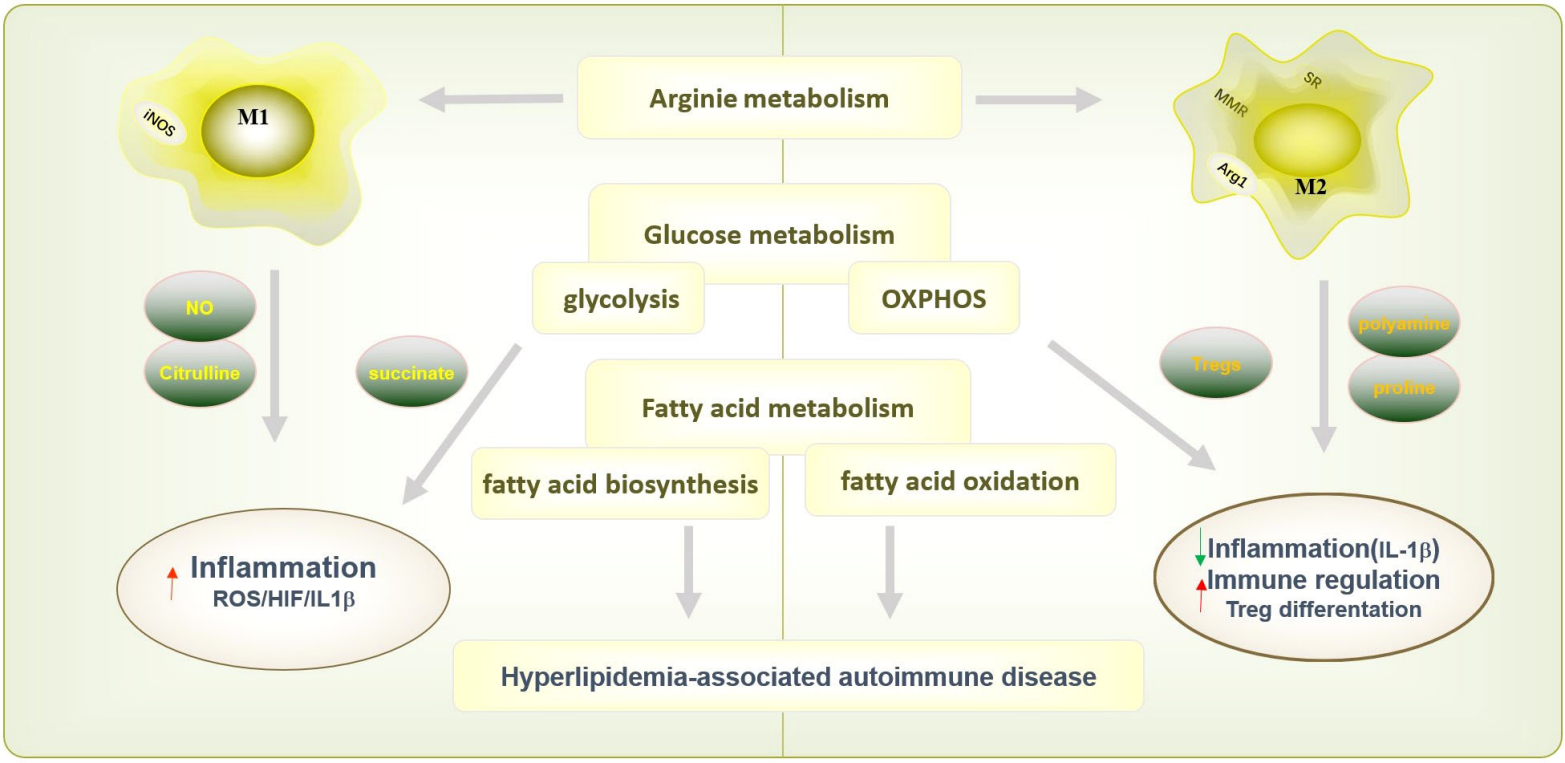
Macrophages polarization and function in metabolism processes. Macrophage are also involved in a variety of metabolic processes, including arginine metabolism glucose metabolism and fatty acid metabolism, to exert different immunological functions. The M1 macrophages express nitric oxide synthase (NOS) to metabolize arginine into NO and citrulline, which further promotes inflammatory response. Instead, M2 macrophages inhibited inflammation by medicating polyamine/proline synthesis. M1 macrophages carry out glycolysis, and causes succinate accumulation and promote inflammation by ROS/HIF-1α/IL1β pathway. And M2 macrophages carry out oxidative phosphorylation (OXPHOS) could decrease IL-1β after pro-inflammatory activation in macrophage, and promoted Treg differentiation to increase the regulatory function in immune system. M1 macrophage rely on fatty acid biosynthesis and M2 macrophage require fatty acid oxidation. The function of macrophage on fatty acid metabolism modulates inflammatory signatures and involved in hyperlipidemia-associated autoimmune diseases include psoriasis, RA, and SLE.

Macrophages are also involved in a variety of metabolic processes, including arginine metabolism and glucose metabolism, which was indicated in **Figure 2**. The M1 macrophages express nitric oxide synthase (NOS) to metabolize arginine into NO and citrulline, which further promotes the synthesis of downstream active nitrogen, finally facilitating inflammatory response (50, 51). In addition, M2 macrophages regulate arginine metabolism and thus regulate cell proliferation, tissue repair and inhibited inflammation by medicating polyamine/proline synthesis (52). Macrophages maintain adaptive responses to oxygen gradients and hypoxia by regulating their glucose oxidative phosphorylation, glycolysis and fatty acid oxidation (53). Based on the demands for energy and the production of specific functional-associated factors, pro-inflammatory macrophages and anti-inflammatory macrophages opt for distinct metabolic pathways upon activation. Instead of M2 macrophage, M1 macrophages carry out glycolysis and rely on fatty acid biosynthesis, and increased glycolysis causes succinate accumulation and promote inflammation by ROS/(hypoxia-inducible factor-1α) HIF-1α/IL1β pathway (54, 55). On the other hand, M2 macrophages possess a high basal mitochondrial oxygen consumption rate (OCR), carry out oxidative phosphorylation (OXPHOS), and require the induction of fatty acid oxidation (55, 56). Different fatty acid metabolism, particularly mitochondrial fatty acid oxidation in macrophage modulates inflammatory signatures and macrophage phenotype, which indicated the vital function of macrophage in hyperlipidemia-associated autoimmune diseases include psoriasis, RA, and SLE. Programmed macrophages by setting metabolic commitment for OXPHOS increased programmed death ligand 1 (PD-L1) expression, decreased IL-1β after pro-inflammatory activation in macrophage, and promoted Treg differentiation to increase the regulatory function in immune system (57). Moreover, macrophages resist parasite infection by regulating glutathione and redox metabolism and participate in tissue repair, tumor growth and anti-inflammatory response by regulating iron metabolism (58–60). Furthermore, the metabolites, in turn, mediate the macrophage response to inflammation. Recent study has found that citrulline levels in lipopolysaccharide (LPS) and IFNγ-stimulated macrophages are significantly reduced, which promotes inflammatory signals by activating Janus kinase 2 (JAK2)- signal transducer and activator of transcription 1 (STAT1) pathway (61). Citrulline can inhibit bacterial load in the spleen and liver of Listeria monocytogenes-infected mice by impeding pro-inflammatory macrophage activation (61).

### 2.5 Macrophage polarization

Macrophages display specific phenotypes and rapidly change their functions under the local microenvironment, called macrophage polarization (62). The phenotypes of macrophage polarization are generally divided into two types: one is classically activated macrophages (M1), which are pro-inflammatory and involved in the elimination of pathogens and resist infection. The other is alternative activation macrophages (M2) that are anti-inflammatory and involved in tissue repair and reconstruction (63). The Th1 cytokine, such as IFNγ or LPS, can induce M1 polarization, while Th2 cytokines, such as IL-4, can induce M2 polarization. Intracellular metabolite profiles of each macrophage activation state presented a unique metabolic signature. The 1D 1H NMR-based metabolomics identified increased adenosine triphosphate (ATP) and decreased intracellular nicotinamide adenine dinucleotide (NAD^+^) in M1 macrophage, and increased adenosine diphosphate (ADP), guanosine triphosphate (GTP), adenosine monophosphate (AMP) in M2 macrophage (64). The M1 macrophages express high levels of pro-inflammatory cytokines, active nitrogen and oxygen intermediates, promote the responses of Th1 and Th17 by secreting IL12 and IL23, and have strong bactericidal and tumor-killing activity (63, 65). However, the M2 macrophages indicate high phagocytic activity and high expression of scavenger receptor (SR), macrophage mannose receptor (MMR), arginase-1(Arg-1), IL10, TGF-β, which are mainly involved in parasite containment, phagocytosis, promote tissue repair, wound healing, angiogenesis, fibrosis and immune regulation (66, 67). In fact, depending on induced agents, expressed markers, secreted mediators and functions, M2 macrophages are further classified as M2a, M2b, M2c, as well as M2d macrophages. The M2a macrophages induced by IL-4 or IL-13, also known as wound healing macrophages, can increase endocytosis activity and have immunity to parasites, tissue repair, collagen formation and fibrogenesis (68). M2b macrophages stimulated by immune complexes, TLR ligands or IL-1β, also known as regulatory macrophages, have strong anti-inflammatory and immunosuppressive effects (69). M2c macrophages induced by glucocorticoids, IL-10 or TGF-β promote phagocytosis and clearance of dead cells (70). M2d macrophages induced mainly by TLR antagonists, also known as tumor associated macrophages (TAM), can promote angiogenesis and tumor progression (71). However, M1 and M2 macrophages are the two extremes of the activation state of macrophages which cannot fully represent macrophages in the complex microenvironment *in vivo*. The dynamic balance of M1/M2 is crucial to maintain homeostasis. Response to foreign stimulation such as microbial infection or tumor, M1 macrophage is activated and promote inflammation to perform robust antimicrobial and anti-tumoral function. And to protect against the chronic inflammatory response, M1 macrophage is inhibited by regulatory mechanisms driven by anti-inflammatory function of enhanced M2 macrophages differentiation and promote tissue regeneration, angiogenesis and wound healing (72). And the imbalance contributes to the occurrence and development of many diseases including infection, tumor and autoimmune diseases (73–75). Fortunately, the high degree of plasticity allows macrophage switch from one phenotype to another depending on encountered micro-environment signals in each specific tissue, which providing a potential treatment target for autoimmune disease.

## 3 Macrophages in SLE

**Figure 3:**
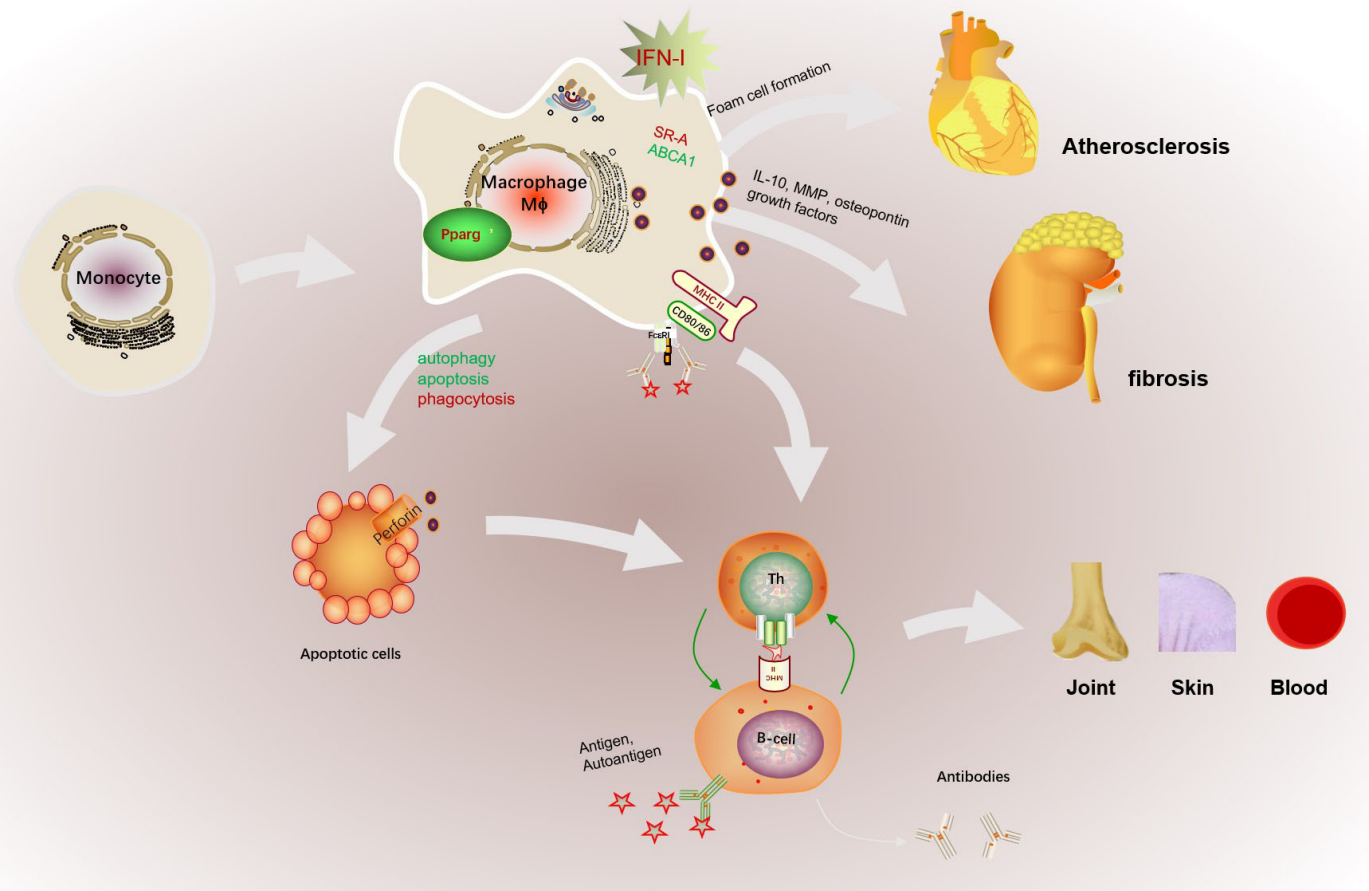
The abnormal activation of macrophage in SLE. The abnormal microenvironment in SLE patients, such as the high expressed IFN-I, promotes monocyte to recruit and differentiate into macrophages. In addition, increased antigen presentation of macrophage promotes B cell activation assisted with Th cells and further promotes the production of autoantibodies. Macrophages can also cause cardiovascular risk by promoting foam cell formation with increased SR-A and decreased ABCA1 expression. The dysfunction of macrophage phagocytosis may increase the gathered apoptotic cells and results in retention of apoptotic cell debris in SLE patients. Increased macrophage apoptosis and autophagy could contribute to autoantibody formation and organ damage by increased apoptotic load and impaired clearance of apoptotic material, finally exacerbated the production of autoantigens. Besides, macrophage infiltration the kidney promoted glomerular cell proliferation and early fibrosis by IL-10, MMP, osteopontin and growth factors. Besides, increased apoptotic cells serves as autoantigen to aggravate autoimmune reaction and may cause multiple targeted organs such as skin, joint, or blood.

SLE is a chronic systemic autoimmune disease with diverse clinical manifestations characterized by immune system infiltration and inflammation in damaged organs covering skin, lungs, joints, kidneys and central nervous system (76). The abnormalities in the activation state of circulating and tissue macrophages in patients with SLE are crucial factors in the occurrence and development of the disease (77, 78). Depleting macrophage attenuated skin and kidney disease severity, which suggested the vital function in SLE pathogenesis (79, 80). The pro-inflammatory patrolling monocytes (PMOS) accumulated in the glomeruli in SLE patients and lupus mice are the main components of lupus glomerular or kidney inflammation (81). Emerging evidence has demonstrated that macrophage infiltration is associated with lupus nephritis in mice and humans (82, 83). Renal macrophage infiltration appears in spontaneous NZB/W nephritis and IFN-accelerated models of lupus nephritis (84). The function and numbers of MZMs are also reduced in autoimmune BXD2 mice (85). The absence of MZMs results in retention of apoptotic cell debris within the marginal zone and drives follicular Ag-transportation by marginal zone B (MZB) cells to stimulate an autoimmune response (85). The abnormal functions of macrophage in SLE are indicated in **Figure 3**.

The phagocytic ability of macrophages from SLE patients is weakened, which results in the production of autoantibodies and SLE-like autoimmune nephropahy (78, 85). Hence the dysfunction of macrophage phagocytosis may partly explain the gathering of apoptotic cells in the germinal center of lymph nodes in SLE patients (86). The mechanism response to the reduced clearance rate of macrophages has been widely demonstrated. It has been shown that the absence of PPARγ in macrophage cannot obtain an anti-inflammatory phenotype in the presence of apoptotic cells, finally resulting in glomerulonephritis and the autoantibodies production of nuclear Ags (87). The transcription factors Kruppel-like factor 2 (KLF2) and KLF4 also control apoptotic cells clearance program in tissue macrophage and maintain the homeostasis (88). Moreover, the increased autophagy and apoptosis in macrophage also contribute to the pathogenesis of SLE. The autophagy-related genes (*Atg5*, *Atg12* and *Beclin 1*) were significantly upregulated in the splenic and renal macrophages in activated lymphocytes-derived DNA (ALD-DNA) induced lupus mice and in the peripheral blood mononuclear cells from SLE patients. And adoptive transfer of autophagy-suppressed macrophages alleviated lupus symptoms in SLE mice (89). Increased monocyte/macrophage apoptosis could contribute to autoantibody formation and organ damage by increased apoptotic load and impaired clearance of apoptotic material, finally exacerbated the autoimmune phenotype in NZB x SWR lupus-prone mice (90). Besides, research about lupus nephritis in NZB/W mice suggested that macrophage infiltration in the kidney promoted glomerular cell proliferation and early fibrosis by IL-10, MMP, osteopontin and growth factors (91).

SLE is a prototype autoimmune disease in which genetics play a major role. Researchers have identified many new loci which are attributed to the pathogenesis of SLE by genome­wide association studies (GWAS). SLE susceptibility loci related to macrophages are mainly concentrated in genes that affect type I interferon (IFN­I) signaling, NF­κB activation, TLR signaling, phagocytosis and immune tolerance. Currently, more than 100 genetic risk sites related to SLE and more than half of them are closely related to the production or response of IFN­I (92). IFN­I promotes monocyte differentiation and the expression of MHCII and costimulatory molecules (such as CD40, CD80 and CD86) of macrophages to promote T cell activation (93, 94). Besides, increased IFN­I levels in SLE patients can further promote the recruitment and adhesion of monocytes, and accumulation of macrophages in kidney and vascular lesions of SLE patients (95–97). In addition, IFN­I enhances scavenger receptor SR-A and reduces ATP binding cassette subfamily A 1 (ABCA1) expression to promote cholesterol efflux, oxidation low lipoprotein (ox-LDL) uptake in macrophage and foam cell formation, which increasing the risk of cardiovascular diseases (96). Abnormal increased IFN­I promotes the translocation of MZB cells to the follicular region of the spleen and disrupts the interaction between MZBs and MZMs, preventing clearance of apoptotic cells debris and follicular entry deterrence of apoptotic cells by MZMs (98, 99). The amplified TLR7 signaling in macrophage activation during antiviral responses and autoimmune diseases can occur product IFN­I in turn by promoting phosphorylation and activation of MAP kinase p38 and transcription factor STAT1 (100). *TNIP1*(TNFAIP3-interacting protein 1, also known as ABIN1), a characteristic susceptibility gene for SLE identified by GWAS can regulate IFN-I production in DCs and macrophages through the TLR7 pathway (101). Large numbers of renal myeloid cells in patients with lupus nephritis, including macrophages, are activated. Almost all known susceptibility genes that affect innate immune signals may potentially affect the progression of lupus nephritis by activating myeloid cells in the kidney (102). Some genes such as *ITGAM* and *FCR* can potentially affect the recruitment of myeloid cells to the glomerular matrix by binding to the immune complexes in the glomerulus (103). DCs, macrophages and endothelial cells engulf C1q-coated apoptotic cells, and deficient in the complement protein C1q inhibit the clearance of apoptotic material and intensify lupus-like skin manifestations in mice and humans (100). *ITGAM* is an established SLE susceptibility locus, which impairs phagocytosis of complement-opsonized targets in monocytes, neutrophils and macrophages. In conclusion, these susceptible genes promote SLE pathogenesis through IFN-I-macrophage immune axis, and rebalancing macrophage functions may resist the damage of highly expressed IFN-I.

Abnormal macrophage polarization also has been identified in the occurrence and development of SLE. The overwhelming M1 macrophages promote the exposure of autoantigens and the occurrence of autoimmune reactions (104, 105). The gene expression profiles of myeloid cells from active SLE patients expressed higher M1-related genes and tend to promote inflammation. In comparison, myeloid cells from inactive SLE patients expressed higher M2-related genes and participated in immune repair (106). Aberrantly expanding M1 macrophages were dominating in MRL-Fas(Lpr) mice, hastened the onset of lupus nephritis, mediated defective renal repair and non-resolving inflammation (107). In the early stage of apoptosis, M2 macrophages can promote the production of anti-inflammatory factors and phagocytize apoptotic cells in an anti-inflammatory way called “bubble drink” (108, 109). Increased M2 macrophages reduced pro-inflammatory cytokines expression and increased the secretion of anti-inflammatory cytokines, which could be used for anti-inflammatory therapy in SLE (110). TIPE2 overexpression by AAV-TIPE2 induced M2 macrophage polarization, induced serum anti-dsDNA autoantibody and pathological renal damage, increased urine protein levels in the ALD-induced SLE mice (111). Adoptive transplantation of M2 macrophages or stimulating monocytes to differentiate into M2-like macrophages significantly reduced the severity of SLE, while M1 macrophage metastasis aggravated the development of SLE (112, 113). Virgin olive oil and its phenolic components have been shown to prevent various inflammatory and immune diseases, which may be related to inhibiting M1 and promoting M2 macrophage polarization (114, 115). The above studies show that the abnormal polarization of macrophages plays a vital role in SLE, which will be a potential target for SLE therapy.

Current therapies for SLE are designed to resolve inflammation with the goal of preventing permanent organ injury, and reduce clinical symptoms. Mycophenolate mofetil (MMF), an inhibitor of purine synthesis, inhibits the recruitment of monocytes and the production of nitric oxide and superoxide in activated macrophages to restrain tissue damage (116). The heterogeneity of disease mechanisms in SLE suggests that cell- and cytokine- or pathway-specific therapies for macrophage would be effective in treatment for SLE.

## 4 Macrophages in RA

RA is an autoimmune disease characterized by chronic inflammation that eventually results in joint damage and even joint dysfunction. It has been found that macrophage infiltration is positively correlated with the degree of joint erosion, and increased synovial macrophage infiltration in synovial tissue is an early sign of RA (117–119). Clodronate could reduce knee swelling, inflammation and joint destruction by eliminating synovial macrophages in rats with antigen-induced arthritis (AIA) (120). Various mechanisms generally lead to increased macrophage infiltration in inflammatory sites, such as facilitating the expression of chemokines and pro-inflammatory cytokines, local survival rate/reducing apoptosis (121). Inhibited macrophage infiltration in synovial tissue may be a protential target for RA treatment. Increased apoptosis of Ly6C^+^ monocyte derived macrophages, reduced monocyte migration into the ankles and enhanced macrophage migration from the inflamed synovial tissue to the draining lymph nodes are responsible for the reduction of macrophages in synovial tissue after infliximab treatment alleviated disease progress in hTNF-Tg mice (122).

**Figure 4:**
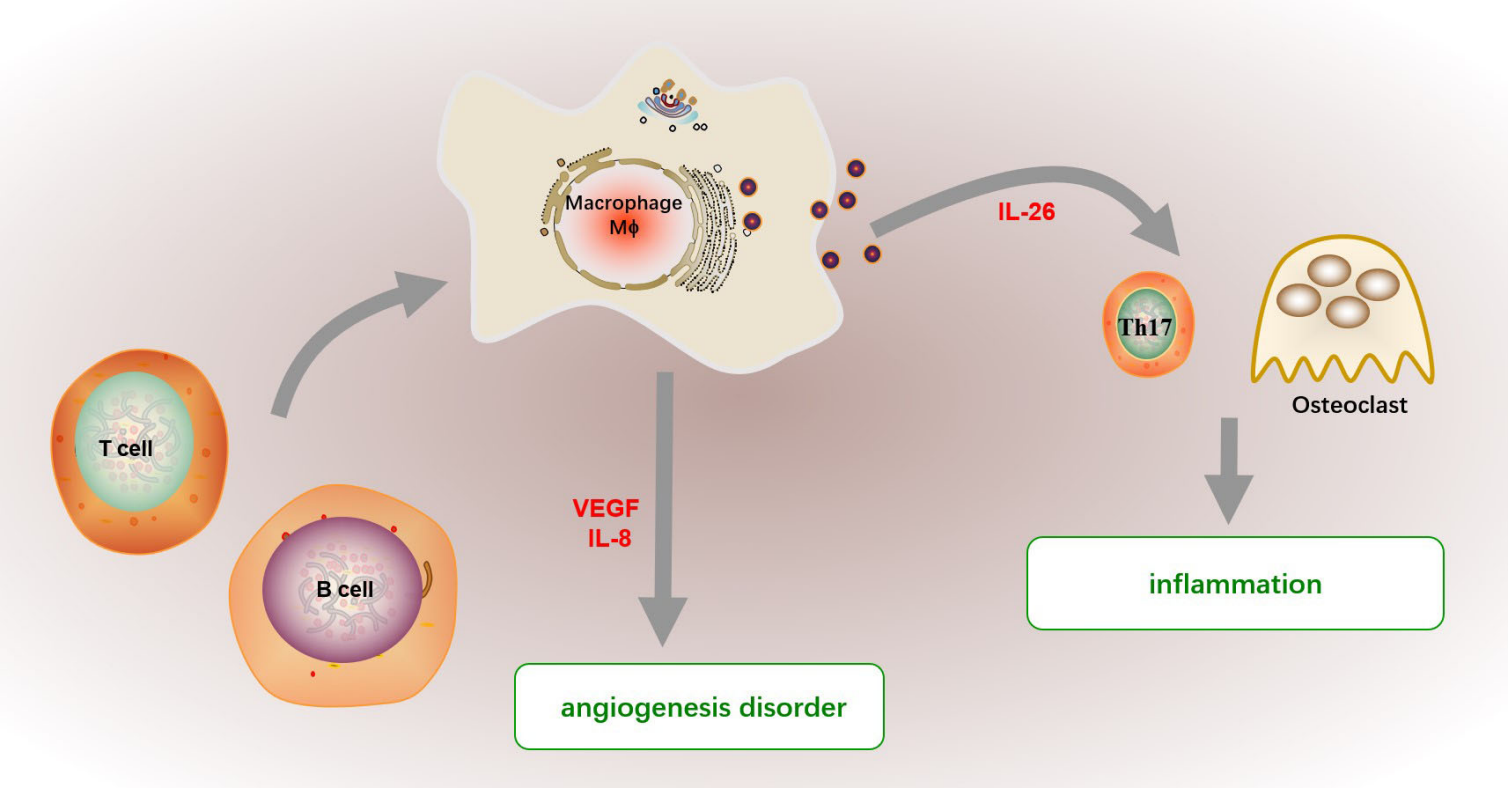
The function of macrophage in RA. The infiltrated macrophage further mediated various inflammatory cell states in synovitis by orchestrating cytokine network. Macrophages promote inflammation by promoting Th17 cell differentiation and stimulating osteoclast differentiation by secreting cytokines including IL-26. Besides, macrophages in synovial tissue and fluid mediate the chemotaxis and proliferation of endothelial cells, promote the formation of pannus and infiltration of inflammatory cells, and further expand the inflammation in RA by producing endothelial growth factor (VEGF). And macrophage-derived IL-8 also promote angiogenesis disorder in RA. The abnormally activated macrophage in RA patients show a proinflammatory profile, which may be supported by activated B or T cells.

The infiltrated macrophage further mediated various inflammatory cell states, significantly contributing to the initiation and perpetuation of synovitis in RA by orchestrating cytokine network (123), as shown in **Figure 4**. Macrophages expedite inflammation by promoting the production of Th17 cells and stimulating osteoclast differentiation by secreting cytokines including IL-26 (124, 125). Besides, macrophages in synovial tissue and synovial fluid mediate the chemotaxis and proliferation of endothelial cells, promote the formation of pannus and infiltration of inflammatory cells, and further expand the inflammatory response in RA by producing vascular endothelial growth factor (VEGF) (126, 127). And macrophage-derived IL-8 also promote angiogenesis disorder in RA (128). The mechanism of abnormal activation of macrophages is not clear at present. Burbano. et al. found that increased circulating microparticles (MP) forming immune complexes in SLE and RA patients favored the polarization of monocyte-derived macrophages into a proinflammatory profile, which promoted T and B cell activation, and B-cell survival (129). Transcriptome profiles of highly inflamed RA synovial tissue (RA-ST) also demonstrated that monocytes/macrophages show similar gene patterns induced by bacterial and fungal, and activated B or T cells also activate monocytes/macrophages (130).

Current conventional synthetic and biologic disease-modifying anti-rheumatic drugs (DMARDs) used in the clinic to treat of RA are related to adjusting macrophage activation and reducing synovial macrophage infiltration. Methotrexate, leflunomide or sulfasalazine reduces macrophage accumulation by promoting apoptosis and inhibiting Th1 response (123). Besides, the anti-TNF biological anti-rheumatic drugs such as etanercept, adalimumab decreased inflammatory cytokines production and increased phagocytosis in monocyte derived macrophages, which all alleviated inflammatory reactions (131). In addition, various monoclonal antibodies targeting biomolecules produced by macrophages are available for the therapeutic options of RA. The therapeutic efficacy of blocking granulocyte-macrophage colony stimulating factor receptor (GM-CSF) pathway like anti-GM-CSFR monoclonal antibody mavrilimumab is linked to inhibited production of pro-inflammatory mediators such as VICM (citrullinated and MMP degraded vimentin fragment) biomarker released by activated macrophages (132, 133). A monoclonal antibody to folate receptor β (FR-β) produced by macrophages specifically accumulates in inflamed lesions of murine RA and peritonitis disease models, facilitating immune cells, including T cells, B cells, neutrophils and DCs, to exit from the inflamed lesions and allative disease processes (134).

The imbalance of macrophage polarization also occurs in RA. The blood monocytes from RA patients had a propensity for preferential differentiate toward M1-like macrophages that contributed to synovial inflammation (135). Transcriptional omics study showed that synovial macrophages facilitate the expression of pro-inflammatory genes (*INHBA, FCER1A, SLC2A1, MMP12, EGLN3, NOS* and *CCR2*) but restrain anti-inflammatory genes (*IGF1, HTR2B, FOLR2* and *CD36*) expression (136, 137). Besides, M1 macrophages are characterized by decreased heme uptake and iron output but increased iron storage, which could partly explain the phenomenon of anemia in RA patients (138). The M1-to-M2 macrophage re-polarization can also serve as a promising treatment for RA. Targeted biologics that selectively regulate the function of macrophages have broad research prospects for the treatment of RA and also could solve the adverse effects of non-targeted drugs to a certain extent. Interfering with glycolytic pathways activated in M1 macrophages can reduce pro-inflammatory factors production and IgG antibodies, finally alleviating joint inflammation and damage in CIA mice (139). The administration of Wilforlide A reduced clinical scores, joint swelling and histological damage of collagen-induced RA mice by inhibiting the secretion of pro-inflammatory factors (MCP1, GM-CSF and M-CSF) and iNOS in the synovium (140). Angiotensin II type 2 receptor (AT2R) activation and a developed triamcinolone-gold nanoparticle (Triam-AuNP) complex promotes proinflammatory synovial macrophages to differentiate into the tolerogenic macrophage, finally attenuating the joint pathology in a rat model of collagen-induced RA (141, 142). The above studies have shown that M1 macrophages are dominant in RA synovium, regulating abnormal macrophage polarization is one of the important therapies of RA. Many new drug vectors and targets have been found to regulate macrophage function selectively. Encapsulated plasmid DNA encoding IL-10 and the chemotherapeutic drug betamethasone sodium phosphate (BSP) in biomimetic vector M2 exosomes derived from M2 macrophages, folate-modified triptolide liposomes (FA-TP-Lips) and folic acid modified silver nanoparticles(FA-AgNPs) all serve as a promising biocompatible drug to facilitate M2 macrophages polarization selectively, thereby treating RA safely and effectively (143–145). However, macrophages are incredibly heterogeneous. The focus and difficulty of RA drug development will be how to distinguish, identify and act on specific activated pathogenic macrophages.

## 5 Macrophages in SSc

**Figure 5:**
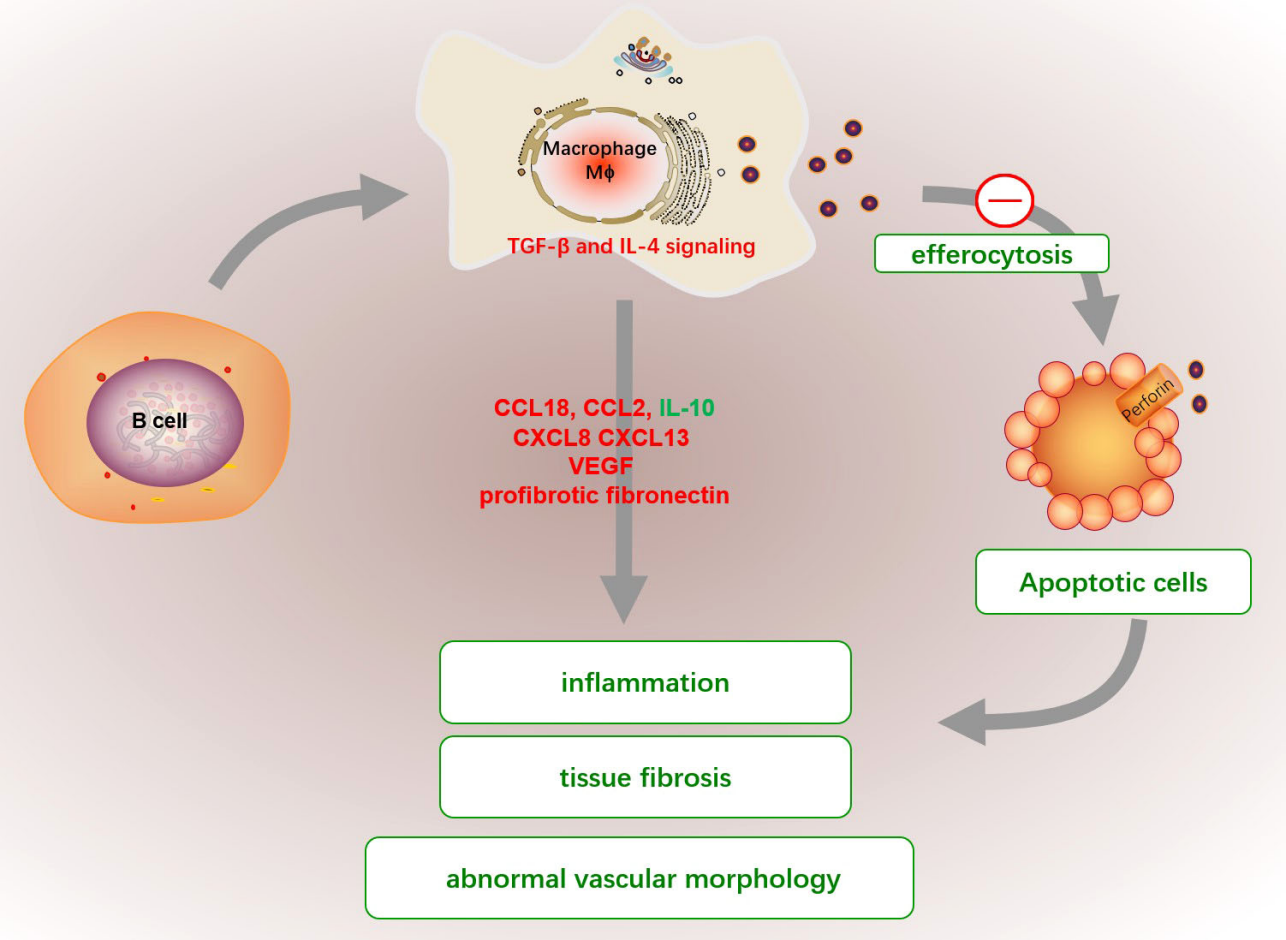
The abnormal functions of macrophage in SSc. The efferocytosis capacities of macrophage in SSc patients are significantly reduced, which cause the emergence of circulating nuclear antigens and promote proinflammatory fibroblasts. Activated macrophages produced a variety of cytokines, such as high levels of CCL18, CCL2, and CXCL8 but low IL-10 expression, which enriched in perivascular regions of highly fibrotic SSc skin to favor pro-inflammatory fibroblasts. Additionally, the excessive production of CXCL13, elevated profibrotic fibronectin and VEGF by macrophages can also promote tissue fibrosis, immune activation and abnormal vascular morphology in SSc. The fibrotic macrophage might be activated by a dysfunctional B cell or dysregulation of TGF-β and IL-4.

Systemic sclerosis (SSc) is a chronic multi-system disease characterized by autoimmunity, immune cell infiltration and activation, fibrosis and vascular lesions, often accompanied by skin involvement and visceral dysfunction including heart and lungs caused by fibrosis (146, 147). Vascular complications such as pulmonary hypertension and scleroderma renal crisis have become the leading causes of disability and death of SSc (148, 149). The infiltrating inflammatory leukocytes in the new affected skin from SSc patients are mainly CD14^+^ monocytes/macrophages (150). Transcriptomics analysis found that monocytes continuously migrated and differentiated into alveolar macrophages to promote fibrosis during pulmonary fibrosis and selectively targeting the differentiation of alveolar macrophages in the lung may improve fibrosis (151). These researches suggested that monocytes/macrophages play an essential role in the early pathogenesis of SSc, which was displayed in **Figure 5**.

Apoptotic cell clearance (efferocytosis) capacities of monocyte-induced macrophage from SSc patients are significantly lower than those in healthy donors, which partly explains the emergence of circulating nuclear antigens (152). Besides, macrophage is a main contributor for fibirosis. The CD14^+^ monocytes and CD14^+^ pulmonary macrophages in SSc patients have elevated profibrotic fibronectin production and are considered extracellular matrix producers (153). Activated macrophages produced a variety of cytokines, such as high levels of CCL18, CCL2, and CXCL8 but low IL-10 expression, which enriched in perivascular regions of highly fibrotic SSc skin to favor pro-inflammatory fibroblasts (154, 155). Additionally, the excessive production of CXCL13 and vascular VEGF by macrophages can also promote tissue fibrosis, immune activation and abnormal vascular morphology in SSc (156, 157). The formation mechanism of fibrogenic macrophages is still unclear. It has been demonstrated that fibrotic macrophage might be activated by a dysfunctional B cell in mice with bleomycin-induced SSc, and correlated with the severity of fibrosis in SSc patients (158). Besides, Dysregulation of TGF-β and IL-4 signaling may also be responsible for the pro-fibrotic function in SSc macrophages (159).

The abnormal polarization of macrophages in SSc is relatively complex. Studies have found that crystalline silica SiO or response gene to complement 32 (RGC32) can promote macrophages to form an M1-like phenotype and reduce M2 polarization, which caused the reduction of macrophages efferocytosis in SSc (160, 161). However, the gene expression profiles of affected skin, lung, esophagus and peripheral blood in patients with SSc showed that the expression of M2-related genes was significantly up-regulated in macrophages with pronounced fibrogenic effect (162). Infiltrated macrophages in skin lesions from SSc and local scleroderma were found to highly express CD163 (163–165), indicating that M2 macrophage may also involve in skin fibrosis. Besides, studies have found that some biological agents can inhibit the process of SSc by reversing the polarization of M2 macrophages. The PDE4 inhibition induced by nintedanib, rolipram and apremilast and glycyrrhizin all ameliorate the fibroblast activation by impeding M2 macrophage function in SSc-related mice (166–168). All the above studies indicate that M2 macrophage infiltration may be a target for SSc treatment. However, researchers had found the number of M1 and M2 macrophages in the skin of SSc patients was significantly increased, indicating that macrophages in different polarized states might synergistically promote the pathogenesis of SSc (169). Skin biopsy RNA examined by next-generation RNA sequencing suggested that most early diffuse SSc patients had a concomitant M1 and/or M2 macrophage signature, suggesting co-occurrence of dysregulated fibroblast and macrophage polarization (169). Studies about TLR signaling in fibrosis in SSC and other fibrotic diseases hinted that the conflicting results may be related to long-term inflammatory stimulation (170). Furthermore, macrophages can acquire memory-like characteristics to copy with antigen exposure, protection against re-infection and more efficient vaccine strategies. Recent research found that trained macrophage acquired memory-like characteristics in response to antigen exposure can be targeted to SSc treatment. Low-dose LPS training and adoptive transfer alleviated fibrosis and inflammation in SSc mice, while BCG-training aggravated disease in this model (171). The long-term and complex *in vivo* microenvironment may be an essential promoter of macrophage activation that is unique to SSc patients. However, the function and mechanism need to be further explored.

## 6 Macrophages in T1D

T1D is an autoimmune disease characterized by the continuous destruction of islet cells caused by islet leukocyte infiltration (172). The loss of pancreatic β cells can lead to uncontrolled blood glucose and various complications such as cardiovascular disease, nephropathy, retinopathy, heart attack and stroke, which require lifelong dependence on exogenous insulin (173). Islet inflammation is one of the main mechanisms of pancreatic β-cell injury and the development of T1D. In diabetes-prone biological breeding rats (DP-BB), it has been demonstrated that macrophages are the first immune cells to infiltrate into islets (174). Furthermore, there were no lymphocytes in the islets when macrophage infiltration was prevented (175), suggesting that lymphocyte recruitment in islets depends on the macrophage. In addition, the immunohistochemical results of pancreatic specimens from newly diagnosed T1D patients confirmed the presence of macrophages in early and advanced inflammation (176). Various research about spontaneous T1D animal models has shown that specific clearance of macrophages *in vivo* can significantly inhibit Th1 but increase Th2 immune response induced mainly by IL-12, and inhibited cytotoxic effector of CD8^+^ T, even remaining selective acceleration of the recruitment of CD8^+^ T cells into the islets (177–179). Depleting macrophage by liposomes containing clodronate also selectly abolished diabetogenic CD4^+^ T cells induced diabetes even with inflammation existence (180).

The microenvironment in T1D pancreas promote the recruitment of macrophages and abnormal functions. It was found that islet resident macrophages of non-autoimmune mice had immunomodulatory phenotype and could promote Treg cell differentiation *in vitro* (181). Deficiency of immunomodulatory function in macrophages may be an essential mechanism of pathogenesis of T1D (181). In addition, the migration and phagocytosis to target inflammatory cells of macrophage in the streptozotocin (STZ) -induced T1D model weakened islet cell immune defense (182). Diabetgenic CD4 T cells produce a variety of inflammatory cytokines and chemokines such as CCL1, resulting in the recruitment of macrophages into pancreas (183). Reduced integrin-associated surface factor CD47 on islet cells promoted macrophage migration and phagocytosis of endogenous cells (182). Instead of clearing apoptotic cells silently without production of pro-inflammatory cytokines, macrophages in T1D secret inappropriately high amounts of IL-1β and TNF-α to contribute to the initiation or continuation of an immune attack towards the pancreatic beta-cells (184). Besides, previously research also showed that macrophages from non-obese diabetic (NOD) mice are activated and engulf apoptotic cells at a lower rate, which might result in secondary necrosis, inflammation and self-antigen presentation in T1D (185).

**Figure 6:**
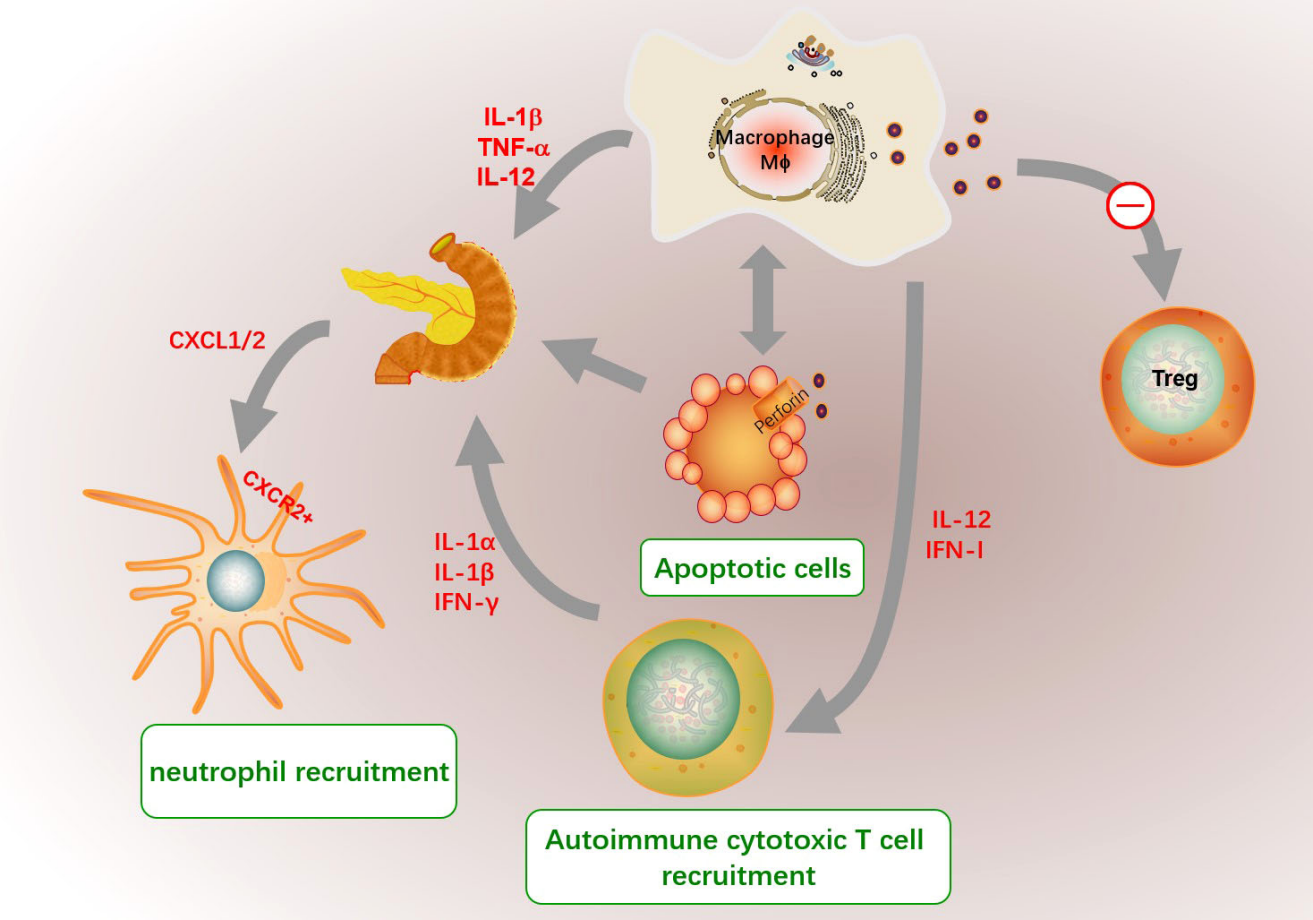
The role of macrophages in the pathogenesis of T1D. The microenvironment in T1D pancreas promote the recruitment of macrophages and abnormal functions. Islet resident macrophages in T1D had defected immunomodulatory phenotype and might inhibit Treg cell differentiation. In addition, the migration and phagocytosis to target inflammatory cells such as apoptotic cells of macrophage in T1D are decrease. And macrophage secret inappropriately high amounts of IL1β and TNFα to contribute to the initiation of an immune attack towards the pancreatic beta-cells. The unengulfed apoptotic cells might result in secondary necrosis, inflammation and self-antigen presentation in islet. And increased macrophage-derived cytokines including IL-12, TNF-α and IL-1β are responsible for the inflammatory cascade of events leading to the destruction of pancreatic β cells. The interaction between inflammatory macrophages and β-cells promote the production of CXCR2 ligands (CXCL1 and CXCL2), which further recruit diabetogenic CXCR2^+^ neutrophils. Autoreactive CD4^+^ T cells destroyed β cells assisted by cytokines IL-1α, IL-1β, and IFN-γ. Besides, macrophage derived IL-12 and IFN-I signaling might contribute to the development and activation of β cell–cytotoxic Th1 and CD8 cells.

And increased macrophage-derived cytokines including IL-12, TNF-α and IL-1β selectively in spleen lymphocytes and pancreatic islet are responsible for the inflammatory cascade of events leading to the destruction of pancreatic β cells (186). Macrophages are involved in regulating the infiltration and functions of immune cells in T1D. Recruited macrophages in the pancreas by diabetes-derived T cell produce IL-1β, TNF-α and NO, and express chemokine receptors CCR5, CXCR3 and CCR8 to further recruit and active other inflammatory cells (183). The interaction between inflammatory macrophages and β-cells promote the production of CXCR2 ligands (CXCL1 and CXCL2) in the pancreas of T1D mice, which further recruit diabetogenic CXCR2^+^ neutrophils from the blood into the pancreatic islets (187). Autoreactive CD4^+^ T cells destroyed β cells through a Fas-dependent mechanism that was assisted by cytokines IL-1α, IL-1β, and IFN-γ (188). Besides, macrophge derived IL-12 might contribute to the development and activation of β cell–cytotoxic Th1 and CD8 cells in NOD mice (189). And macrophages selectively traffick autoimmune cytotoxic T cells into the islets *via* IFN-I signaling even without entering the islets, and ablation of IFN-I signaling on macrophages limits the onset of T1D (190). The role of macrophage on the pathological process of T1D was shown in **Figure 6**.

Macrophage polarization may act as a potential therapeutic agent for T1D. M2 macrophages explicitly located in the inflammatory pancreas could significantly inhibit the proliferation of T cells and promote the survival of β cells after adoptive transfer into spontaneous T1D mice, resulting in resistance to T1D in non-obese resistant (NOR) mice (191). In addition, the survival of transplanted islets was partly dependent on the content of M2 macrophages (192, 193). The early glycosylation products (EGPs) produced in the first step of Maillard reaction/glycosylation alleviated insulin resistance and pancreatic immune infiltration by increasing the M2/M1 ratio (194). Macrophage-specific knockout ubiquitin coupling enzyme E2 can weaken the energy metabolism and M2 type polarization of macrophages, thus increasing the risk of diabetes T1D induced by STZ (195). Hence, promoting M2 but inhibiting M1 macrophage polarization may be an important target for preventing and treating T1D.

Macrophage-derived proinflammatory cytokines, chemokines and their receptors were identified the suitable targets for the therapeutic interventions of T1D. The TNF-α inhibitor infliximab could alleviate T1D, which might be related with the reduced presentation of islet antigen to both effector CD4^+^ and CD8^+^ T cells (196, 197). And IL-6 has also been suggested as a target for T1D treatment (198). Multiple strategies blocking the CXCR1/2 pathway main expressed in macrophage inhibited leucocyte recruitment and prevent inflammation and autoimmune mediated islet damage, which was new interventional approach for T1D (199).

## 7 Discussion

The possible functions of macrophages in autoimmune diseases as described in **Table 1**. In brief, the scavenging ability of macrophages was destroyed, leading to the accumulation of autoimmune complexes in local tissues. Besides, the abnormal macrophage activation induced a series of irrepressible pro-inflammatory responses, and promoted the activation and recruitment of lymphocytes in local tissues, resulting in tissue damage. In addition, the aberrant polarization of macrophages has been identified to contribute to the pathogenesis of autoimmune diseases. However, due to the significant heterogeneity of macrophages, the polarization of macrophages varies significantly in different tissues and even in different phases of the same disease. Systematically and comprehensively understanding the polarization of macrophages in autoimmune diseases will conduce to the prevention and treatment of autoimmune diseases.

**Table 1:** Possible function of macrophages in autoimmune diseases.

	Macrophage function	Macrophage polarization
SLE	• Increased autophagy of macrophage • Aggravating the inflammatory response • Reduced phagocytosis to apoptotic cells	↑ M1: Increasing secretion of pro-inflammatory cytokines ↓ M2: Reducing anti-inflammatory factors and phagocytize apoptotic cells
RA	• Increased anti-apoptosis • Increaed migration of macrophages to local tissues • Promoting Th17 differentiation and joint inflammation • Promoting the formation of pannus and causes further infiltration of inflammatory cells	↑ M1: Increasing iron storage and glycolysis, releasing pro-inflammatory cytokines to promote inflammation
SS	• Increased anti-apoptosis in macrophage • Reduced phagocytosis to apoptotic cell • Producing multiple cytokines to participate in fibrosis and angiogenesis	↑ M2: Increasing M2-related fibrotic phenotype
T1D	• Promoting the inflammatory response in islets • Mediating T cells recruitment and activation • Promoting immune cell infiltration and autoimmune response in islets	↓ M2: Insulin resistance and pancreatic immune infiltration are related to reduced M2 polarization

Currently anti-macrophage therapy in autoimmune diseases mainly focuses on down-regulation the production of abnormal macrophage-derived pro-inflammatory cytokines production, elimination of dysfunctional macrophage from the inflammatory regions such as inhibiting monocyte recruitment and differentiation, and upregulation of anti-inflammatory cytokines. In recent years, macrophage-derived extracellular vesicles composed of microvesicles and exosomes have aroused increased interest in the treatment for autoimmune disease. The macrophage-derived extracellular vesicles are considered as optimal delivery vehicles for the minimal toxicity and specific target effect. Macrophage-derived microvesicle-coated poly (lactic-co-glycolic acid) (PLGA) nanoparticles to encapsulate tacrolimus significant suppress the progression of RA in mice, which is an efficient biomimetic vehicle for RA targeted treatment (200). Besides, macrophage-derived extracellular vesicles efficiently delivered dexamethasone into inflamed kidney and effectively suppress inflammation and fibrosis in kidney (201).

Various new techniques such as single-cell sequencing, metabolomics and other multi-omics research methods have been applied in autoimmune diseases research and have achieved considerable achievements. A single-cell sequencing result of a mixed lung cell sample from bleomycin-induced lung injury mice found a group of disease-related transitional macrophages that specifically express CX3CR1 and PDGF-AA and are located in fibrotic scars to promote fibrosis (202). This study provides an effective target for preventing and treating pulmonary fibrosis-related diseases. In addition, single-cell pseudo-time analysis infer the transcription trajectory of macrophages when they gradually change their gene expression profile during autoimmunity, suggesting that we can find the molecular changes in the early stage of the disease and the most decisive target. The application of multi-omics methods at the single-cell level will provide an effective means for exploring the potential mechanisms of abnormal macrophage phenotypes and offer a solid theoretical basis for preventing and treating autoimmune diseases.

## Author contributions

SY wrote the main manuscript. SJ, MZ revised the manuscript. SJ had primary responsibility for the final content. All authors agree to be accountable for the content of the work. All authors contributed to the article and approved the submitted version.
